# Communicating prognosis to women with early breast cancer – overview of prediction tools and the development and pilot testing of a decision aid

**DOI:** 10.1186/s12913-019-3988-2

**Published:** 2019-03-15

**Authors:** Viktoria Mühlbauer, Birte Berger-Höger, Martina Albrecht, Ingrid Mühlhauser, Anke Steckelberg

**Affiliations:** 1 MIN Faculty, Health Sciences and Education, University of Hamburg, Martin-Luther-King Platz 6, D-20146 Hamburg, Germany; 2 Institute for Health and Nursing Science, Martin Luther University Halle-Wittenberg, Magdeburger Str. 8, D-06112 Halle, Germany

**Keywords:** Decision-making, Decision support techniques, Prognosis, Breast neoplasms, Risk communication, Prediction tool

## Abstract

**Background:**

Shared decision-making in oncology requires information on individual prognosis. This comprises cancer prognosis as well as competing risks of dying due to age and comorbidities. Decision aids usually do not provide such information on competing risks. We conducted an overview on clinical prediction tools for early breast cancer and developed and pilot-tested a decision aid (DA) addressing individual prognosis using additional chemotherapy in early, hormone receptor-positive breast cancer as an example.

**Methods:**

Systematic literature search on clinical prediction tools for the effects of drug treatment on survival of breast cancer. The DA was developed following criteria for evidence-based patient information and International Patient Decision Aids Standards. We included data on the influence of age and comorbidities on overall prognosis. The DA was pilot-tested in focus groups. Comprehension was additionally evaluated through an online survey with women in breast cancer self-help groups.

**Results:**

We identified three prediction tools: Adjuvant!Online, PREDICT and CancerMath. All tools consider age and tumor characteristics. Adjuvant!Online considers comorbidities, CancerMath displays age-dependent non-cancer mortality. Harm due to therapy is not reported.

Twenty women participated in focus groups piloting the DA until data saturation was achieved. A total of 102 women consented to participate in the online survey, of which 86 completed the survey. The rate of correct responses was 90.5% and ranged between 84 and 95% for individual questions.

**Conclusions:**

None of the clinical prediction tools fulfilled the requirements to provide women with all the necessary information for informed decision-making. Information on individual prognosis was well understood and can be included in patient decision aids.

**Electronic supplementary material:**

The online version of this article (10.1186/s12913-019-3988-2) contains supplementary material, which is available to authorized users.

## Background

Women with breast cancer want to be involved in treatment decision-making [1–3]. The German guideline on breast cancer [4] and the German National Cancer Plan [5] recommend shared decision-making (SDM), which includes the consideration of women’s values and preferences. Especially the National Cancer Plan emphasizes the importance of patients’ abilities to ask for understandable information and support in order to move from a traditional, paternalistic to an active, equal patient-physician relationship. In addition, studies have shown that gender has a substantial effect on health behaviors, access to health care, and health system responses. In some settings and conditions women’s health is more negatively affected [6]. So far, SDM has not been widely implemented in clinical practice [7].

In order to make an informed treatment decision, patients and clinicians need to know about the risk of dying from breast cancer. However, it is just as important to consider the individual prognosis [8]. Howlader et al. used the term individual prognosis in contrast to cancer prognosis. Individual prognosis does not only include the risk of dying from cancer, but also the risk of dying from competing risks other than cancer. These competing risks are age or comorbidities. For younger women, there are hardly any differences between cancer prognosis and individual prognosis since the risk of dying due to other causes than cancer is low. For older women, for example with cardiovascular or kidney diseases, the risk of dying from breast cancer might be small compared to the risk of dying from their comorbidities. Knowledge about these comorbidities is essential in order to estimate the benefit and harm of additional chemotherapy. If the baseline risk of dying from other causes than breast cancer is high, only very few women might benefit from chemotherapy. Therefore, patient characteristics like age, comorbidities and age-dependent risks of death from causes other than breast cancer have to be considered in estimating the individual prognosis [8]. The information should be made available to physicians and patients in prediction tools and DAs. So far, we are not aware of any DA or patient treatment guideline including data on individual prognosis.

The aim of the project “specialized nurses to support informed shared decision-making in oncology” (the acronym “SPUPEO” refers to the German translation) is to improve cancer care by offering decision-coaching. Decision-coaching is led by breast care nurses or oncology nurses to enhance SDM to allow women to make informed choices. An informed choice is achieved when women’s preferences are *congruent* with the intention and implementation of their decision. Evidence-based patient information (EBPI) is a prerequisite for informed decision-making. Detailed criteria for EBPI have been published [9, 10]. Evidence-based DAs communicate benefits and harm of all treatment options including the option not to treat, presenting them in a way that patients can understand. In contrast to EBPI, DAs additionally contain value clarification tools. The SPUPEO decision-coaching is supported by evidence-based DAs that have been developed within the project. SPUPEO involved members of self-help groups from the very beginning of the process. These women contributed to the selection of topics, resulting in two priority topics: the treatment decision for ductal carcinoma in situ (DCIS) [11] and for early, hormone receptor-positive breast cancer. Results on the development and pilot-testing of the DCIS intervention with emphasis on coaching by specialized nurses have recently been published [12]. In the present study, we focus on the communication of individual prognosis among women facing decisions on the treatment for early, hormone receptor-positive breast cancer. It comprises endocrine therapy only or a combination of endocrine and chemotherapy. The SPUPEO DA quantifies the possible treatment benefits and harm. It also includes information on the natural course of the disease without any drug treatment.

To estimate the benefit from adjuvant therapy, several online prediction tools have been developed [13]. Usually, prognosis is based on pathological prognostic factors like tumor size, grading and number of positive lymph nodes. In recent years, various biomarker tests have been available to predict whether a woman will benefit from chemotherapy in addition to endocrine therapy [14–17]. However, none of the biomarker tools considers baseline mortality risks and the influence of comorbidities. They might therefore falsely evoke an impression of certainty regarding the treatment decision.

The manuscript consists of two parts. The aim of this study is to describe the development and pilot-testing of the DA for early, hormone receptor-positive breast cancer including information on individual prognosis. We started with a literature search on prediction tools addressing individual prognosis in order to be able to communicate information on individual prognosis in the DA. In the first part of the following sections we will therefore give an overview on prediction tools that formed the basis of the development process. The second part comprises the development and piloting of the DA.

## Methods

### Prediction tools: An overview

This part gives an overview of existing online prediction tools for women with breast cancer.

In January 2016, a systematic literature search for interactive, freely accessible prediction tools concerning breast cancer was performed in PubMed and the Cochrane Controlled Trials Register. We searched for tools addressing the effect of adjuvant pharmacotherapy on survival based on clinical parameters (tumor and patient characteristics) and checked for updates on a monthly basis until January 2019. Tools were analyzed for included variables, information on prognosis without adjuvant therapy, underlying data bases, model validation and being up-to-date. Publications in languages other than English or German were excluded, as were studies published before the year 2000 (before the first elaborated tool, Adjuvant!Online, was released) as well as tools based on biomarker testing or tools estimating survival through case-matches. Study types eligible for inclusion were meta-analyses, systematic reviews, randomized controlled trials, reviews and comparative studies. Two reviewers (VM, AS) independently screened the titles and abstracts. Disagreements were solved by discussion. Articles were read in full text and checked for eligibility by VM and AS. Data extraction was carried out by VM and AS. Descriptive analysis was performed.

### Decision aid: Development and pilot-testing

This part describes the development and pilot-testing of the DA, including information on individual prognosis.

As DAs are complex interventions, we developed and pilot-tested the DA in accordance with the UK Medical Research Council’s guidance (phase 1 and 2) [18]. Our results are reported in line with the revised Criteria for Reporting the Development and Evaluation of Complex Interventions in healthcare: revised guideline (CReDECI 2) [19].

### Development of the DA

The development of the DA is based on the theory of planned behavior [20, 21]. We conducted a systematic review of DAs for women with breast cancer. None of the 12 identified DAs concerning chemotherapy for early, hormone receptor-positive breast cancer fulfilled the EBPI criteria [9, 10]: Data on prognosis without drug treatment was usually not available and the evidence underlying the DA was not accessible. We therefore decided to develop a DA according to the EBPI criteria, the Good Practice guidelines for health information [22] and the International Patient Decision Aids Standards (IPDAS) [23]. The development of the DA was comparable to the development of the other SPUPEO DA concerning DCIS [12].

We undertook a systematic literature search in March 2014 to identify the evidence base related to the following topics:

- use of chemotherapy regimens recommended in the German treatment guideline [4] (taxanes, anthracyclines, CMF (cyclophosphamide, methotrexate, 5-fluorouracil)) in adequate dosing
- use of endocrine therapy as recommended in the German treatment guideline (tamoxifen, aromatase inhibitors, GnRH-analogues) in adequate dosing and treatment period (at least 5 years of endocrine therapy)
- direct comparison of endocrine therapy plus chemotherapy versus endocrine therapy alone
- endocrine therapy versus placebo to estimate the natural course of the disease

Additionally, we searched medical guidelines (national and international) and further references from professional associations. Studies concerning neoadjuvant chemotherapy were excluded.

We checked for updates on a monthly base until January 2019. Study types eligible for inclusion were meta-analyses, systematic reviews and RCTs. We included observational studies for side effects if the data from RCTs were insufficient. We searched PubMed, Embase and the Cochrane Controlled Trials Register (search strategy see Additional file 1 p.2). Publications in languages other than English or German were excluded. Two reviewers (VM, MA) independently screened titles and abstracts. Disagreement was solved by discussion. Articles were read in full text and checked for eligibility by VM and BBH. In addition, we searched for grey literature and screened the references of large meta-analyses by the Early Breast Cancer Trialists’ Collaborative Group (EBCTCG). All included studies were critically appraised regarding risk of bias using the Cochrane risk of bias tool [24]. Data extraction was carried out by two independent reviewers (VM, BBH). Descriptive analysis was performed. The DA was developed as a brochure. To allow good readability, we chose a large font size (14.5pt) and format (21x29cm). Due to the topic, we used female designations throughout the brochure. The content of the DA can be found in the supplement (see Additional file 1 p.3).

### Pilot-testing

User testing was carried out using focus groups and an online survey. The results were reported according to the Consolidated Criteria for Reporting Qualitative Health Research (COREQ) [25]. All the interviewers were health scientists from Hamburg University and had experience in conducting focus group interviews. The interviews were carried out at the meeting places of self-help groups and at Hamburg University. The methodological orientation to underpin the study was the theory of planned behavior.

#### Focus groups

The aim of the focus group study was to explore the acceptability, comprehensibility and completeness of the DA. Focus groups aim to find the range of opinions of people across groups. They present a more natural environment compared to individual interviews [26]. For the focus group study, we recruited women from breast cancer self-help groups in northern Germany via e-mail. Women were eligible to participate if they had any type of invasive breast cancer and could read the DA in the German language. They received the DA two weeks before the interview. After having given informed consent, the focus group interviews were conducted by two interviewers (VM and AS/MA/BBH) based on an interview guide. All focus group interviews were audio taped, and in addition, notes were taken. We conducted a content analysis according to Mayring [27]. One researcher coded the transcripts and the results were discussed by two researchers. The results guided the revision in an iterative process. All focus group interviews were carried out between January and September 2016. All the women received a compensation of 15€.

The group discussions focused on prognosis considering age and comorbidities. Furthermore, uncertainty concerning applicability of data on the benefit of additional chemotherapy was discussed. Focus groups were conducted in an iterative process. Resulting revisions were incorporated and discussed in the following focus group until data saturation was achieved (focus group interview guide see Additional file 1 p.4).

#### Online survey

The presentation of data concerning the influence of age and comorbidities on 5-year survival is expectably difficult to understand. During the focus group interviews, this chapter had been revised several times. To confirm that the information is well understood, a subsequent online survey addressing a larger group of women was conducted from April to August 2017. Women were recruited from breast cancer self-help groups and breast cancer internet forums. We used Unipark® [28] to carry out the online survey. The two relevant pages of the DA were displayed, and participants were asked four multiple choice questions. Two questions tested whether participants were able to derive correct numbers from the Table (Q1a and Q1b), two questions tested comprehension (Q2 and Q3). We analyzed the proportion of correct responses out of the total responses, for all questions individually and for overall questions.

### Ethics statement

The SPUPEO project was approved by the ethics committees of the German Federation of Nursing Science (DPG).

## Results

### Prediction tools: An overview

Out of 19,634 references, 19,585 were excluded based on their titles and abstracts. Forty-nine full texts were screened and 36 fulfilled the inclusion criteria.

We identified three relevant prediction tools concerning the effect of adjuvant pharmacotherapy on survival: Adjuvant!Online [29], PREDICT [30, 31] and CancerMath [32]. CancerMath address health professionals only. All tools are freely accessible, however Adjuvant!Online has been unavailable since the end of 2015. All descriptions are therefore based on the former version. Seven other tools for women with breast cancer were identified but excluded from the overview [13, 33–38] (see Additional file 1 p.1).

### Characteristics of included tools

#### Pathological prognostic factors

Adjuvant!Online, PREDICT and CancerMath consider tumor characteristics like tumor size and grading, number of positive nodes, estrogen-receptor (ER) status and human epidermal growth factor receptor 2 (HER2) status. PREDICT also considers mode of detection (screening or symptomatic) and Ki67 status. CancerMath considers the histological type.

#### Patient characteristics

All tools consider age at diagnosis. Only Adjuvant!Online considers comorbidities, even though precise definitions of the comorbidity stages provided are not available. CancerMath displays age-dependent non-cancer mortality. PREDICT has included competing mortality in its algorithm and links it to age. Differences in vitality and functional status within age groups are not considered.

#### Presentation of results

All tools display disease progression without adjuvant therapy after surgery. Adjuvant!Online and CancerMath differentiate between cancer death and death from other causes. CancerMath displays survival rates, cancer death rates and non-cancer death rates. Data are available for up to 15 years. If hormonal therapy and chemotherapy are selected, only the additive effect of both treatments is presented. The effect of HER2-targeted therapies is not displayed. Effects are presented as curves, bar charts, pie charts or pictograms. PREDICT shows overall survival at five and ten years in bar charts. Additional effects of endocrine therapy, chemotherapy and trastuzumab are presented separately. Non-cancer mortality is not available. Neither PREDICT nor CancerMath provides estimates on risk of recurrence, but Adjuvant!Online does.

#### Harm

Communicating the harm caused by chemotherapy is one of the challenges for physicians. Harm is influenced by many factors including age, comorbidities and drug interactions. If the expected benefit is small, data on harm becomes even more relevant. None of the tools considers harm that might be caused by pharmacotherapy. In a cohort study of 12,239 women with breast cancer [39], receiving chemotherapy was associated with a higher risk of hospital admissions or emergency room visits within the first year after breast cancer diagnosis (61% with chemotherapy versus 42% without chemotherapy, difference: 19% [95% CI 16.7 to 21.3%, *p* < .001]). Chemotherapy patients had longer hospital stays (5.0 versus 3.8 days, difference: 1.2 [95% CI 0.6 to 1.7]) and 16% of women who received chemotherapy had a chemotherapy-related serious adverse event compared to 5% of women who did not receive chemotherapy (difference: 11% [95% CI 9.6 to 12.4%]). In this study, chemotherapy recipients and non-recipients did not differ in comorbidity score, but recipients were younger and more likely to have metastatic disease. In the group of women receiving chemotherapy, hospital admission rates varied according to age and chemotherapy regimen [40], a fact that is insufficiently displayed by all prediction tools.

#### Underlying data, actuality and funding

Adjuvant!Online is based on the Surveillance, Epidemiology and End-Results (SEER) registry from the American National Cancer Institute. PREDICT uses the Eastern Cancer Registration and Information Centre (ECRIC) dataset, containing UK data. CancerMath is based on the SNAP method (size, nodes, and prognostic factors) which was developed using data from women treated in Southern California, the Surveillance, Epidemiology and End-Results (SEER) registry from the American National Cancer Institute and National Vital Statistics Reports.

Information on funding is rarely reported. PREDICT is partly financed by the pharmaceutical industry. Adjuvant!Online was developed in 2001 and was shut down for updates in 2015. There is no information about a relaunch of the website. PREDICT was launched in 2010. Since then, updates led to the inclusion of HER2 status, Ki67 and micrometastases. It was updated and refitted in 2017 to improve calibration. It seems that CancerMath is currently not being updated. Since all the tools are based on registers and provide an estimation of the prognosis for 10–15 years, it follows that all the tools are usually based on data and therapies that were introduced more than 10 years ago. Therefore, transferability is questionable.

#### External validation

All prediction tools were externally validated.

##### Adjuvant!Online

We identified 13 studies [30, 41–52] conducted in different Western and Asian populations. The differences between predicted and observed survival varied substantially among subgroups.

##### Predict

Ten studies [30, 31, 48, 51–57] from different Western populations were retrieved. Ten-year survival was accurately predicted apart from several subgroups; five-year survival was significantly overestimated.

##### CancerMath

We identified two studies [32, 58]. The participants were grouped according to risk of death (1–2%; 2–4% etc.). Predicted and observed survival rates were conform for 97% of the study population.

Further details are summarized in Table 1.

**Table 1:** External validation of prediction tools

Author	Validation cohort	Results
**Adjuvant!Online**		
Campbell et al. (2009) (version 8.0) [41]	1065 patients ≤85 years with T1–2, N0, M0 tumors diagnosed between 1986 and 1996 at Churchill Hospital in Oxford (UK).	For the whole cohort at 10 years, Adjuvant!Online **significantly overestimated** OS (by 5.54%, *P* = 0.001), BCSS (by 4.53%, P = 0.001), and EFS (by 3.51%, P = 0.001). OS significantly overestimated for the following parameters: age, menopausal status, Grade 2 + 3, nodal involvement, tumor size 1–2 cm, ER+, local therapy, no systemic therapy and hormone therapy only.
de Glas et al. (2014) (version 8.0) [42]	2012 patients ≥65 years with early breast cancer diagnosed between 1997 and 2004 in the western Netherlands.	Adjuvant!Online **significantly overestimated 10-year OS.** The difference between observed and predicted 10-year overall survival was 9.8% ([95% CI 5.9–13.7], *p* < 0.0001, c-index 0.75). 10-year cumulative recurrence was overestimated by 8.7% ([95% CI 6.7–10.7], p < 0.0001, c-index 0.67) when comorbidity was defined as “average for age”. Definition of comorbidity by an expert panel resulted in **significant underestimation of 10-year OS** by − 17.1% ([95% CI − 21.0 to − 13.2], p < 0.0001, c-index 0.70) but accurate prediction of cumulative recurrence (− 0.7% [95% CI − 2.7–1.3], *p* = 0.48, c-index 0.62).
Hajage et al. (2011) (version 8.0) [43]	I. 456 French patients with N0 M0 tumors diagnosed between 1995 and 1996. II. 295 Dutch patients with T1–2 N0 M0 tumors ≤52 years diagnosed between 1984 and 1995.	I. No significant difference between predicted and observed survival, but survival overestimated for women receiving chemotherapy only. II. 10-year **OS was significantly overestimated** by 13% (*p* = 0.00001).
Jung et al. (2013) (version 8.0) [44]	699 Korean patients with T1–3, N0–3, M0 treated between 1986 and 1999.	Adjuvant!Online **significantly overestimated 10-year OS** by 11.1%, BCSS by 11.6% and EFS by 9.3% (all *p*< 0.001).
Yao-Lung (2012) (version 8.0) [45]	559 Taiwanese patients treated between 1992 and 2001with N0–3, M0	No significant differences in predicted OS in low-risk patients but overestimation of survival in high risk patients (predicted:observed risk = 1.26; *p* = 0.016)
Mook et al. (2009) (version 8.0) [46]	5380 patients with T1–3, M0 tumors diagnosed between 1987 and 1998 at the Netherlands Cancer Institute.	For the whole cohort, there were no significant differences between predicted and observed 10-year OS and BCSS. **OS was significantly overestimated** for women < 40 years and ≥ 70 years. **BCSS was significantly underestimated** for women with mastectomy, DCIS, and ER-. Tumor size and age resulted in overestimation as well as underestimation of BCSS. C-index was 0.70 for OS and 0.71 for BCSS.
Olivotto et al. (2005) (version 5.0) [47]	4083 patients with T1–2, N0–1, M0 tumors diagnosed between 1989 and 1993 in British Columbia (Canada).	No significant differences between predicted and observed 10-year OS, BCSS and EFS. **OS was significantly overestimated** for women < 35 years, with positive nodes and a combination of hormones and chemotherapy. **OS was underestimated** for women with negative nodes, without systemic therapy and BCS + RT.
**PREDICT**		
Candido Dos Reis (2017) (version 2, refitted) [57]	5738 patients diagnosed between 1999 and 2003 in the UK (ECRIC dataset) 1944 patients diagnosed between 1989 and 1998 from the Nottingham/Tenovus Breast Cancer Study (NTBCS) 981 patients < 50 years from the Breast Cancer Outcome Study of Mutation Carriers (BCOS) diagnosed between 1990 and 2000 with stage I-III breast cancer in the Netherlands. 2609 patients diagnosed between 2000 and 2008 in the UK (POSH dataset)	PREDICT significantly **overestimated ACM** in the POSH dataset by 12% (p = 0.00) and **BCSM** by 9% (*p* = 0.018). Non-breast cancer mortality was significantly **overestimated** by 57% (*p* < 0.001) in the POSH dataset and significantly **underestimated** by 19% (*p* = 0.039) in the NTBCS dataset. Across all datasets, PREDICT significantly **overestimated BCSM** in ER+ women aged 20–29 years by 40% (*p* = 0.0047) and in ER+ and ER- women with tumor size ≥5 mm by 35 and 33%, respectively (*p* = 0.04 and p = 0.00). **BCSM** was significantly **underestimated** in ER+ with tumor size 0-9 mm by 35% (*p* = 0.024). Discrimination was better for ER+ than ER- in all datasets (ER+: AUC from 0.741 in BCOS to 0.796 in ECRIC, ER-: AUC from 0.632 in BCOS to 0.726 in ECRIC).
de Glas et al. (2016) (version 2) [53]	2012 patients ≥65 years with early breast cancer diagnosed between 1997 and 2004 in the western Netherlands.	5-year OS was **underestimated** in patients without comorbidity (predicted:observed OS = − 3.7%, [95% CI = − 7.2 to − 0.2], *P* = 0.040), and **overestimated** in patients with 4 or more comorbidities (predicted:observed OS 11.8%, [95% CI = 6.9–16.7], *p*< 0.0001). 10-year OS was **overestimated** in patients with 4 or more comorbidities (predicted:observed OS = 20.7%, [95% CI = 15.8–25.6]). Overall, c-index of the predicted 5-year OS was 0.73, [95% CI = 0.70–0.75], and for 10-year OS 0.74, [95% CI = 0.72–0.76].
Maishman et al. (2015) (version 2) [54]	3000 patients ≤40 years diagnosed in the UK between 2000 and 2008.	PREDICT provided accurate long-term (8- and 10-year) survival estimates for younger women. **Five-year estimates** were less accurate, with the tool **significantly overestimating** survival by 5% overall, and in subgroups of patients with ER+ tumors, grade 2, tumors ≥1 cm or patients receiving a combination of hormone and chemotherapy. OS was also overestimated for patients receiving second and third generation chemotherapy. PREDICT **significantly underestimated** 5-year survival by 25% among patients with ER- tumors and patients receiving trastuzumab. PREDICT **significantly underestimated** 10-year OS in patients with ER- tumors, grade 3, tumors > 5 cm, and in patients receiving chemotherapy alone. C-index was 0.72 vs 0.69 for ER+ vs ER- at 10 years.
Wishart et al. (version 1) (2010) [31]	5468 patients diagnosed between 1999 and 2003 in the UK	5-year OS was **significantly underestimated** by 1.6% (p = 0.004) but no difference between predicted and observed survival at 8 years. C-index was 0.81 for ER+ and 0.75 for ER-.
Wishart et al. (version 3) (2014) [55]	1726 patients diagnosed between 1989 and 1998 in Nottingham (UK).	No significant differences between predicted and observed breast cancer deaths. C-index was 0.77.
Wong et al. (2015) [56]	1480 Chinese, Malay and Indian patients treated between 1998 and 2006 with stage I-III	No significant differences between predicted and observed breast cancer deaths but overestimated OS for patients < 40 years (5-year OS by 6.8% and 10-year OS by 17.2%.). 5-year OS was underestimated for women without nodal involvement by 3.2%, for ER- by 6% and for Her2+ by 6.6%. 10-year OS was overestimated for Her2-negative by 9.9%. C-index for 5-year OS 0.78 [95% CI: 0.74–0.81] and for 10-year OS 0.73 [95% CI: 0.68–0.78].
**Direct comparisons of two clinical prediction tools**		
Engelhardt et al. (2017) [48] Adjuvant!Online (version 8.0) PREDICT (version 1.3)	2710 women < 50 years from the Netherlands with unilateral breast cancer diagnosed between 1990 and 2000	ACM: Adjuvant!Online **significantly underestimated** ACM by − 2% [95% CI: − 3.7 to − 0.3; *p* = 0.02], PREDICT tends to **underestimate** ACM but not significantly (only significant for women ≤35 years, good prognosis (stage 1, T1, N0)). PREDICT overestimated ACM for poor prognosis by 2.6–9.4% (stage 3, T3, N1) and 2.2% for Her 2 positive patients. C-index PREDICT = 0.70, Adjuvant!Online =0.69. BCSM: PREDICT **significantly overestimated** BCSM by 3.2% (95% CI: 0.8 to 5.6; *p* = 0.007). With Adjuvant!Online, there is no difference between predicted and observed BCSM but it **significantly overestimated** BCSM in various subgroups. C-index PREDICT = 0.73, Adjuvant!Online =0.72. For both tools, calibration curves were accurate for women with predicted 20–40% mortality probability.
Hearne et al. (2015) [49] Adjuvant!Online (and NPI)	92 women < 40 years treated in the UK between 1998 and 2007.	No significant difference between predicted and observed survival.
Quintyne et al. (2013) [50] Adjuvant!Online (and NPI)	77 women with early breast cancer treated in Ireland in 2002.	Predicted 10-year OS was 72.9%, while observed OS was 81.8%. NPI prognostic groups did not separate as well (*P* > .05), and the Adjuvant!Online groups separated better (*P* < .05).
Plakhins et al. (2013) [51] Adjuvant!Online (version 8.0) and PREDICT	71 Latvian BRCA-1 patients treated between 2000 and 2008.	Both tools **significantly underestimated OS.** Adjuvant!Online underestimated 10-year OS (predicted:observed − 9.75%; [95% CI = − 13.93 to − 5.57]; *p* < 0.0001) and BCSS (predicted:observed − 8.64%; [95% CI = − 12.88 to − 4.39]; p < 0.0001). PREDICT underestimated 5-year OS (predicted:observed − 6.67% [95% CI = − 10.14 to − 3.19]; p < 0.0001) and 10-year OS (predicted:observed − 10.21%; [95% CI = − 14.93 to − 5.47]; *p* < 0.0001).
Wishart et al. (2011) [52] PREDICT (version 1) Adjuvant!Online (version 8.0)	3140 patients with stage I or II tumors diagnosed between 1989 and 1993 in British Columbia (Canada).	No significant differences in 10-year OS or BCSS. C-index for PREDICT and Adjuvant!Online for OS was 0.709 vs 0.712 and for BCSS 0.723 vs. 0.727 respectively.
Wishart et al. (2012) [30] PREDICT (version 2, “PREDICT+”) Adjuvant!Online (version 8.0)	1653 patients with stage I or II tumors and known Her2 status diagnosed between 1989 and 1993 in British Columbia (Canada).	No statistically significant differences in 10-year OS for Adjuvant!Online, but OS was **underestimated** for PREDICT by 8.8% (*p* = 0.04) and PREDICT+ by 8.4% (*p* = 0.05). In women aged 20–35 years, all models underestimated OS by 32%. 10-year BCSS was **underestimated** by Adjuvant!Online by 14% (*p* = 0.01) but no significantly differences for PREDICT or PREDICT+. In women aged 20–35 years, all models underpredicted survival by 32%. In HER2-positive women, there were no significant differences in predicted and observed OS. There were no significant differences in breast cancer specific deaths with PREDICT and PREDICT+. Adjuvant!Online underestimated survival by 29% (53 vs 75, p = 0.01). Across all risk categories, calibration was good for Adjuvant!Online (goodness-of-fit, *p* = 0.51), and reasonable for PREDICT+ (goodness-of-fit, *p* = 0.042) and Predict (goodness-of-fit, *P* = 0.032)
**CancerMath**		
Chen et al. (2009) [58]	362,491 patients from SEER dataset	Predicted and observed survival agreed within 1% for patients with a chance of death up to 48%, which comprised 97% of the study population. For the remaining 3%, predicted and observed survival rates agreed within 7%.
Michaelsson (2011) [32]	293,576 patients from SEER dataset diagnosed after 1987 24,771 patients diagnosed at the Massachusetts General and Brigham and Women’s Hospitals (Partners dataset) (1968–2007).	Predicted and observed survival agreed within 2% for the 97% of patients with up to a 48% risk of death, while for the remaining 3% of patients with greater than a 48% chance of death, the expected and observed survival values for each group agreed within 7%. Partners dataset: data not reported.

*ACM: all-cause mortality. BCS: breast conserving surgery. BCSM: breast cancer specific mortality. BCSS: breast cancer specific survival. EFS: event-free survival. NPI: Nottingham Prognostic Index. OS: overall survival. RT: radiotherapy*

Laas et al. [59] compared all three tools in a cohort with 965 ER-positive, HER2-negative patients from the US and Canada. Discrimination is characterized using the C-statistic or the receiver operating characteristic (ROC) curve: 0.67 [95% CI 0.63–0.70] for Adjuvant!Online, 0.72 [95% CI 0.69–0.75] for PREDICT and 0.74 [95% CI 0.71–0.77] for CancerMath. All tools might therefore be possibly helpful for discrimination, but only values above 0.75 are considered as clearly useful [60]. Calibration – the average difference between predicted probabilities and observed survival at 10 years was 9.0% [95% CI 6.0–12.0] for Adjuvant!Online, 8.0% [95% CI 5.0–11.0] for PREDICT and 10.7% [95% CI 0.8–18.0] for CancerMath. The correlation between the different prediction tools was 0.85 between Adjuvant!Online and PREDICT, 0.82 between PREDICT and CancerMath and 0.5 between CancerMath and Adjuvant!Online.

#### Implementation

Adjuvant!Online has been shown to improve SDM and to change treatment decisions in a randomized controlled trial with 432 women [61]. In the group using Adjuvant!Online, significantly fewer women with low tumor severity chose adjuvant therapy (58.3% vs. 86.8%). At the same time, more women with high tumor severity (based on tumor size, receptor status and positive nodes) chose adjuvant therapy. The RCT was conducted with an early version of Adjuvant!Online. Another trial [62] showed hardly any significant impact by using Adjuvant!Online compared to a standard pamphlet. Rather, the decision to take adjuvant therapy or not was determined by practice type (academic or community hospital), nodal status and tumor size.

### Decision aid: Development and pilot-testing

#### Development of the DA

##### Evidence synthesis of treatment regimes

Out of 4897 references, 4828 were excluded, based on title and abstract. Sixty-nine references were read in full text. Only four trials compared endocrine therapy directly to chemo-endocrine therapy [63–66], only one trial fulfilled the inclusion criteria [66–68]. In this trial, CMF (cyclophosphamide, methotrexate, 5-fluorouracil) was compared to tamoxifen as endocrine therapy. Since CMF is no longer offered as first line therapy we searched for further studies comparing CMF and tamoxifen to other regimes plus tamoxifen. The only study identified used underdosed chemotherapy and was therefore excluded [69]. Meta-analyses assume an additional 15–20% reduction in mortality from anthracycline-based chemotherapy compared to CMF [70, 71]. However, these assumptions are based on data that also include women with hormone receptor-negative breast cancer or women receiving endocrine therapy over less than 5 years. The effect of anthracycline-based chemotherapy compared to CMF for women addressed in the DA is therefore uncertain and we refrained from displaying direct comparisons between the two regimes. Hence, data from the study comparing CMF and tamoxifen directly were included, even though the use of CMF is not common in breast cancer treatment any more. In designing the DA, we put a lot of effort into the explanation of the strength and limitations of this approach. In addition, data comparing anthracyclines plus tamoxifen versus a combination of anthracyclines, taxanes and tamoxifen was included. The data on the side effects of chemotherapy compared to endocrine therapy was insufficient and was therefore taken from Cochrane reviews, even though they were partly based on women with metastasized breast cancer [72–74].

Tamoxifen was the only endocrine therapy in trials with placebo comparison. Therefore, risks and benefits of aromatase inhibitors were only presented in comparison with tamoxifen [75].

##### Prognosis

**Fig. 1:**
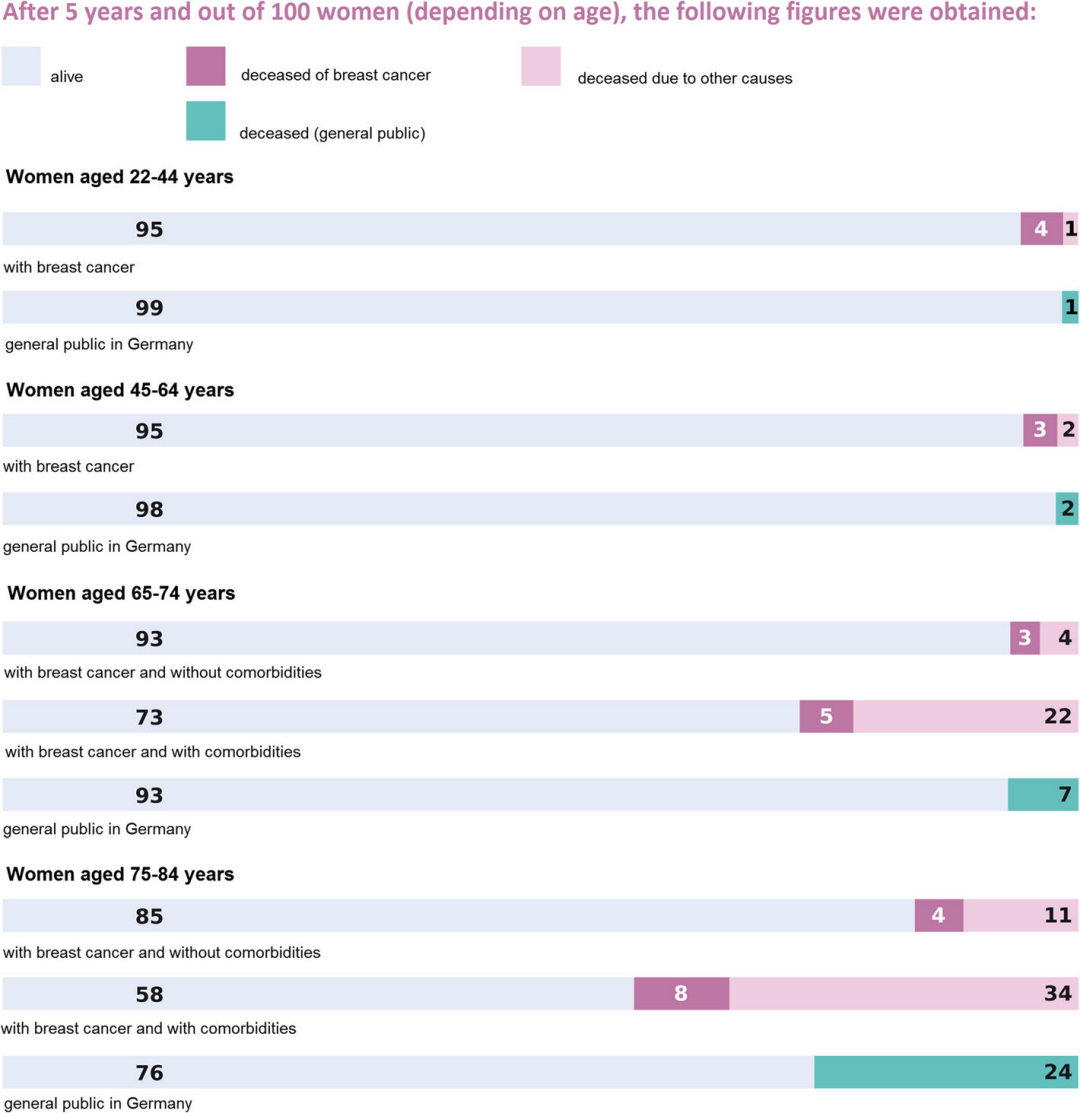
Bar chart on the influence of age and comorbidities on survival from the decision aid

To display individual prognosis rather than cancer specific prognosis only, we provided data on the influence of age and comorbidities on breast-cancer and overall survival (Fig. 1). The underlying data is based on an analysis of SEER registry data [8], even though transferability in non-US populations is uncertain. To set breast cancer patient mortality rates in context and to provide the “baseline” risk of death depending on age, statistical data on the 5-year risk of death in the general population in Germany for different age groups was displayed [76].

#### Pilot-testing

##### Focus group interviews

Four focus groups were conducted including a total of 20 breast cancer patients (1–8 per interview), each interview lasting approximately two hours. The mean age was 60 years (range 32–77). The groups consisted of women with various educational and occupational backgrounds (secondary school nine years: *n* = 5; secondary school ten years: *n* = 7; university entrance diploma: *n* = 8; vocational training: *n* = 10; professional school: *n* = 6; university graduate: *n* = 3; untrained: n = 1). Seven women were currently employed, 19 had received surgery, 16 radiation therapy, 16 endocrine therapy and 13 chemotherapy. When asked about information access, 17 used leaflets and booklets to receive information on health topics, 15 the internet, 13 self-help groups and 12 other media (TV, video, magazines, and newspaper). The frequency of internet use varied: Eight women indicated using the internet on a daily base, five several times a week, four several times per month and three did not use it at all.

The main modifications concerned layout and legend of the bar chart on prognosis, layout and structure of the chapter on chemotherapy and the presentation of side effects.

In general, the DA was found to be helpful, informative and interesting. Some women were pleased and encouraged to be invited to take part in the decision-making process. Further comments from focus group interviews are summarized in Table 2.

**Table 2:** Comments from focus group interviews

Category	Explanation	Quotes
Comprehension	At first sight, the presentation of data from studies using outdated chemotherapy regimens is not comprehensible to women. This view changed during the discussion. The fact that women receive support from decision coaches was highly appreciated and therefore no further modification was necessary.	*“When reading this brochure, I understood what happened to me back then.”* The women comprehended that in certain age groups, *“breast cancer doesn’t account for many deaths”*. *“It cannot be an important piece of information that something is not being used anymore”. “Why should I be interested in this information?”* The information was found to be *“outdated”*, *“of no great importance/relevancy”, “dead weight”. “It might be interesting from a scientific perspective, but it doesn’t help.”* One patient had also the hope “*that recent chemotherapy regimens are better”.* *“I had disposed of this as being too old. However, after the explanation from the experts, it looks very different. But now I find it interesting.” “At first I thought: [data] from the last century? What is that supposed to be? But now I understand. It gives you a clue.”*
Length/extent	The women judged the length of the decision aid differently. They had concerns that the amount of information is too much just after having received the diagnosis. However, the decision aid consists of two parts and only the first part contains the information relevant for making a treatment decision.	*“When I was first flicking through the pages, it didn’t overburden me. With the size of the brochure and the front size, it makes you want to read it.”* *“The structure is very well done. Clearly arranged, everything is well presented so that I as a layperson can understand it.”* *“When you have just received your diagnosis, it is an awful lot to read.”* *“For me, this would have been too much [information].”*
Acceptance	Most of the women appreciated the decision aid and were very happy to be invited to participate in shared decision-making. However, some women were opposed to dealing with content addressing mortality.	*“What a pity that I didn’t have this brochure last year. I found it very good and very helpful.”* *“I didn’t have this information”* *“I can’t say that I knew as much back in 2007 as is written in this brochure.”* *“I wish I had had such a decision aid”.* *“When I read ‘decision aid for women’ I thought: Oh, that’s great, I can take part in decision-making.”* *“You need lots of support and the courage to oppose to the doctor and to say ‘I don’t want this’ (…) and that with brochures like this the inhibition threshold is reduced by saying ‘you’ve got a right to take part in the decision”.* The information on prognosis was found to be “*helpful”, “calming”* and *“lifting up spirits/invigorating”.* *“(I) don’t want to know all this”.* *“I don’t know if I would have wanted to know how many women die.”* *“When I read: ‘death due to breast cancer’ – oh my god, I closed the thing [brochure]. I don’t want to know about that. When I read that, I feel queasy.”*

Two external experts reviewed the DA: One breast cancer activist and one gynecologist.

##### Online survey

Informed consent was provided by 102 women, of whom 86 completed the survey (drop-out rate 16%). Nine participants discontinued when they saw the two pages from the DA, five during the questions and two when asked socio-demographic questions.

The participants completing the survey had an average age of 51 years (27–76 years), 64% had a university entrance diploma, and 26% had graduated from university. All the participants indicated that they spoke German at home. The overall rate of correct responses was 90.5% with ranges between 84 and 95% for individual questions (see Table 3).

**Table 3:** Multiple-choice questions and responses of 86 participants completing the survey

Questions and distractors (correct answer in bold)	Correct responses (n, probability of success % [95% CI])	Incorrect responses (n)	Missing responses (n)
1a: Out of 100 women aged 22–44 years with breast cancer – how many of them are alive after 5 years? (99 / **95**/ 4 / 1)	78, 95% [0.88; 0.99]	4	4
1b: Out of 100 women aged 45–64 years with breast cancer – how many of them have died from other reasons than breast cancer after 5 years? (**2** / 3 / 93 / 96)	73, 89% [0.80; 0.95]	9	4
2: Women aged 65–74 years with breast cancer and other severe comorbidities can die of breast cancer or of other causes. Which statement concerning the risk of dying for these women is correct? **They die more often from other causes than of breast cancer.** They die more often of breast cancer than from other causes. As many women die of breast cancer as from other causes. The graphic does not supply an answer the question.	78, 94% [0.86; 0.98]	5	3
3: Women aged 75–84 years with breast cancer and without comorbidities: Which statement concerning the risk of dying for these women is correct? They die more often of breast cancer than from other causes. As many women die of breast cancer as from other causes. **Despite having breast cancer, less women die compared to the general public.** Women without comorbidities die as often of breast cancer as women with severe comorbidities.	68, 84% [0.74; 0.91]	13	5

*All questions refer to* Fig. 1

## Discussion

The SPUPEO DA for women with early, hormone receptor-positive breast cancer is the first evidence-based DA presenting data on the influence of age and comorbidities on the overall prognosis. We have shown that it is feasible to include data on individual survival in DAs and that women with breast cancer are able to understand the information provided.

Prediction tools can be helpful for SDM [61]. However, none of the identified online clinical prediction tools fulfills the requirements for providing all the necessary information to allow SDM. A major deficit is the lack of information on comorbidities and competing risks of death in order to calculate the individual prognosis and possible treatment benefit. None of the prediction tools considers harm due to adjuvant therapy, either. Several studies have shown the impact of comorbidities on survival [8, 77, 78]. Neither Adjuvant!Online nor PREDICT accurately predicted survival in an elderly cohort with comorbidities. This is probably due to an underrepresentation of elderly patients with functional impairments in the cohort used to develop the model [53]. In the elderly, comorbidity and functionality appear to be more informative for characterizing a patient than chronological age.

### Strengths and limitations

Our study has several strengths. The DA was developed and pilot-tested in accordance with the UK Medical Research Council’s guidance, with EBPI [9, 10] and IPDAS [23] criteria and evaluated in focus groups according to COREQ [25]. It is an important innovation for displaying individual prognosis in a DA for early breast cancer. Results from focus group interviews and the online survey with breast cancer patients show that the information presented is well understood.

The study also has limitations. Data on the influence of age and comorbidities on survival for women with breast cancer are based on US SEER registry data and are therefore just approximations. Besides the uncertainties inherent to all registry data making them only rough estimates, transferability to other countries and healthcare systems is also uncertain. Moreover, our DA addresses women with early hormone-receptor positive, HER2-negative breast cancer whereas SEER data only distinguishes between localized, regional and distant stages of breast cancer.

The DA was developed as a brochure. However, a digital, interactive decision tool is presumably more user-friendly, since patients then only get to see the data concerning themselves. A prediction tool containing data on individual survival is therefore warranted and should be tested in further studies.

The focus group interviews and the online survey were carried out with women from self-help groups. All the women had already made a decision on their cancer treatment and are therefore not in the same situation as the target audience. Moreover, women who get involved in self-help groups have probably better knowledge on cancer therapy and their interest in the topic might be above-average. Still, since women with a broad range of education participated, we believe that the DA is generally easy to comprehend.

Online surveys have a high risk of selection bias. The participants were very well educated, and understanding might differ in other patient samples. However, the DA is part of a concept where specially qualified nurses will support women in their decision-making process [12]. Therefore, women will not be left alone with the information.

#### Meaning of the study results

An accurate prognosis is as essential for SDM as a correct diagnosis, but probably harder to achieve [79]. Appropriate treatment decisions presuppose accurate prognosis, but data thereon are often not available or have severe limitations such as uncertain transferability. As a consequence, physicians have to estimate the prognosis. Presumably, these estimates are often based on intuition and clinical experience only [60]. Data from RCTs would be necessary to show whether estimates based on clinicians’ experience differ significantly from estimates based on algorithms and whether this results in different clinical outcomes. Either way, physicians should also take into account patients’ individual baseline risks. Moreover, they need to consider to which extent prediction tools over- or underestimate benefit and harm in different patient groups. For some patient groups, e.g. with several comorbidities, the database for estimating prognosis may be particularly unreliable. It is within these “grey zones” that the likelihood of an incorrect prognosis arises, resulting in an incorrect starting point for SDM [79]. If physicians use calculators to estimate prognosis and treatment outcomes, they should be aware of discrimination, calibration and how they influence the results [60, 79].

The impact of clinical parameters in prediction tools on prognosis remains unclear. However, risk scores for other diseases - like the Framingham score for coronary heart disease [80] - have shown that prediction tools do not necessarily improve if more parameters are added. Among other tools, Shachar et al. [81] reviewed calculators on life expectancy and chemotherapy toxicity to support SDM in oncology, addressing mainly geriatric patients. Within these tools, the influence of the different variables remains unclear. Rabin et al. [13] reviewed prediction tools for other types of cancer and identified other parameters used to calculate prognosis, e.g. smoking, alcohol consumption or physical activity. However, validation studies are needed to verify their benefit. The same applies to comorbidities. Further research is needed to clarify whether the inclusion of comorbidity status improves prediction [53]. In addition, better data on the impact of comorbidities on the prognosis of long-term survival are urgently needed.

The trend towards biomarker-based decision-making for women with hormone receptor-positive breast cancer concerning the use of chemotherapy [82] is a further challenge due to limited evidence. Recently, the use of chemotherapy depending on genomic testing was investigated in two studies: Cardoso et al. [14] compared risk estimates using Adjuvant!Online and a 70-gene signature for 6693 women diagnosed with breast cancer. In 68%, both instruments estimated risk concordantly (both “low” and “high” risk). In those with discordant risk (high clinical risk and low genomic risk or low clinical risk and high genomic risk), there was no significant difference in 5-year-distant metastasis-free survival and overall survival whether they received chemotherapy or not. For women with high clinical/low genomic risk, refraining from chemotherapy might be disadvantageous: disease-free survival was significantly higher when chemotherapy was administered (93.3% vs. 90.3%, per protocol analysis). Sparano et al. [83] reported a study including 10,273 women with hormone-receptor positive, Her-2 negative, axillary node–negative breast cancer. Patients received a 21-gene expression assay for risk of recurrence. Those with midrange score for risk of recurrence were randomized to receive either endocrine therapy alone or combined endocrine and chemotherapy. At 9 years, endocrine therapy alone was noninferior to chemoendocrine therapy for invasive disease-free survival (invasive disease recurrence, second primary cancer, or death). For many women the benefit of chemotherapy remains unclear, which makes SDM necessary to decide on breast cancer treatment.

Recently, a prediction model based on clinical parameters has been developed to estimate whether biomarker-testing on recurrence is necessary [84]. The model is based on only 6 parameters which were found to be most predictive with grading being the strongest and lymphovascular invasion being the weakest predictor. Individual prognosis and treatment effects were not considered. Further research is needed to find out which parameters and biomarkers in a combined score result in the best prognosis. Overall, the role of biomarker-based decision-making in breast cancer remains unclear.

## Conclusion

Women and physicians need reliable information on prognosis and on how prognosis influences the extent of treatment effects (benefit and harm). Especially data on harm is not incorporated into prediction tools. Data on harm considering comorbidities is hardly available. All tools – whether based on clinical parameters or biomarkers - need to be evaluated in RCTs before they are widely used in clinical practice. For women with breast cancer it is crucial to know what to expect from the different treatment options in order to participate in SDM. Estimates on prognosis, benefit and harm should not be based solely on intuition or clinical experience.

## Additional file

Additional file 1: **Table S1.** 1. Overview of excluded tools for breast cancer patients. Table 1 contains an overview of other tools for breast cancer patients that did not meet our inclusion criteria. 2. Search strategy adjuvant therapy in PubMed. The search strategy listed here was used to search for studies directly comparing endocrine therapy to chemoendocrine therapy in PubMed. 3. Main content of the decision aid. This is the English translation of the table of contents of the decision aid. 4. Interview guide: Focus group interviews SPUPEO DA drug therapy. This is the English translation of the interview guide used for focus group interviews. (DOCX 27 kb)

## Abbreviations

CMF: Cyclophosphamide, methotrexate, 5-fluorouracil
COREQ: Consolidated Criteria for Reporting Qualitative Health Research
CReDECI: revised Criteria for Reporting the Development and Evaluation of Complex Interventions in healthcare
DA: Decision aid
DCIS: Ductal carcinoma in situ
EBCTCG: Early Breast Cancer Trialists’ Collaborative Group
EBPI: Evidence-based patient information
ER: Estrogen receptor
HER2: Human epidermal growth factor receptor 2
IPDAS: International Patient Decision Aids Standards
RCT: Randomized controlled trial
ROC: receiver operating characteristic
SDM: Shared decision-making
SEER: Surveillance, Epidemiology and End-Results registry
SNAP: size, nodes, and prognostic factors
SPUPEO: The acronym “SPUPEO” refers to the German translation of the name of the project “specialized nurses to support informed shared decision-making in oncology”

## References

[1] Hamann J, Neuner B, Kasper J, Vodermaier A, Loh A, Deinzer A (2007). Participation preferences of patients with acute and chronic conditions. Health Expect.

[2] Brown R, Butow P, Wilson-Genderson M, Bernhard J, Ribi K, Juraskova I (2012). Meeting the decision-making preferences of patients with breast cancer in oncology consultations: impact on decision-related outcomes. J Clin Oncol.

[3] Mühlhauser I, Meyer G, Steckelberg A (2010). Patients demand informed participation in medical decision making, but the information data base and structures are not available. Z Allg Med.

[4] Leitlinienprogramm Onkologie (Deutsche Krebsgesellschaft, Deutsche Krebshilfe, AWMF): S3-Leitlinie Früherkennung, Diagnose, Therapie und Nachsorge des Mammakarzinoms, Version 4.0, 2017. AWMF Registernummer: 032-045OL. Available from: http://www.leitlinienprogramm-onkologie.de/leitlinien/mammakarzinom/ [Accessed 04 February 2019].

[5] German Federal Ministry of Health. [National cancer plan – action fields, goals and recommendations for implementation]. 2012. Available from: https://www.bundesgesundheitsministerium.de/themen/praevention/nationaler-krebsplan/der-nationale-krebsplan-stellt-sich-vor.html [Accessed 04 February 2019].

[6] Hawkes S, Buse K (2013). Gender and global health: evidence, policy, and inconvenient truths. Lancet..

[7] Härter M, Dirmaier J, Scholl I, Donner-Banzhoff N, Dierks M-L, Eich W (2017). The long way of implementing patient-centered care and shared decision making in Germany. Z Evid Fortbild Qual Gesundhwes.

[8] Howlader N, Mariotto AB, Woloshin S, Schwartz LM (2014). Providing clinicians and patients with actual prognosis: cancer in the context of competing causes of death. J Natl Cancer Inst Monogr.

[9] Bunge M, Mühlhauser I, Steckelberg A (2010). What constitutes evidence-based patient information? Overview of discussed criteria. Patient Educ Couns.

[10] Lühnen J, Albrecht M, Mühlhauser I, Steckelberg A. Guideline evidence-based health information. 2017. Available from: http://www.leitlinie-gesundheitsinformation.de [Accessed 04 February 2019].

[11] Berger-Höger B, Liethmann K, Mühlhauser I, Haastert B, Steckelberg A (2015). Informed shared decision-making supported by decision coaches for women with ductal carcinoma in situ: study protocol for a cluster randomized controlled trial. Trials..

[12] Berger-Höger B, Liethmann K, Mühlhauser I, Steckelberg A (2017). Implementation of shared decision-making in oncology: development and pilot study of a nurse-led decision-coaching programme for women with ductal carcinoma in situ. BMC Med Inform Decis Mak.

[13] Rabin BA, Gaglio B, Sanders T, Nekhlyudov L, Dearing JW, Bull S (2013). Predicting cancer prognosis using interactive online tools: a systematic review and implications for cancer care providers. Cancer Epidemiol Biomark Prev.

[14] Cardoso F, van’t Veer LJ, Bogaerts J, Slaets L, Viale G, Delaloge S (2016). 70-gene signature as an aid to treatment decisions in early-stage breast Cancer. N Engl J Med.

[15] Paik S, Tang G, Shak S, Kim C, Baker J, Kim W (2006). Gene expression and benefit of chemotherapy in women with node-negative, estrogen receptor-positive breast cancer. J Clin Oncol.

[16] Martin M, Brase JC, Calvo L, Krappmann K, Ruiz-Borrego M, Fisch K (2014). Clinical validation of the EndoPredict test in node-positive, chemotherapy-treated ER+/HER2- breast cancer patients: results from the GEICAM 9906 trial. Breast Cancer Res.

[17] Duffy MJ, McGowan PM, Harbeck N, Thomssen C, Schmitt M (2014). uPA and PAI-1 as biomarkers in breast cancer: validated for clinical use in level-of-evidence-1 studies. Breast Cancer Res.

[18] Craig P, Dieppe P, Macintyre S, Michie S, Nazareth I, Petticrew M (2008). Developing and evaluating complex interventions: the new Medical Research Council guidance. BMJ..

[19] Möhler R, Köpke S, Meyer G (2015). Criteria for reporting the development and evaluation of complex interventions in healthcare: revised guideline (CReDECI 2). Trials..

[20] Ajzen I (1991). The theory of planned behavior. Organ Behav Hum Decis Process.

[21] Ajzen I (2005). Attitudes, personality and behavior.

[22] Arbeitsgruppe GPGI. [Good practice guidelines for health information]. Z Evid Fortbild Qual Gesundhwes. 2016;110–111:85–92. 10.1016/j.zefq.2015.11.005 26875040

[23] Elwyn G, O’Connor AM, Bennett C, Newcombe RG, Politi M, Durand M-A (2009). Assessing the quality of decision support technologies using the international patient decision aid standards instrument (IPDASi). PLoS One.

[24] Higgins J, Green S. Cochrane Handbook for Systematic Reviews of Interventions. Version 5.1.0 [updated March 2011]. The Cochrane Collaboration; 2011. Available from: http://handbook.cochrane.org [Accessed 04 February 2019].

[25] Tong A, Sainsbury P, Craig J (2007). Consolidated criteria for reporting qualitative research (COREQ): a 32-item checklist for interviews and focus groups. Int J Qual Health Care.

[26] Krueger RA, Casey MA (2014). Focus groups: a practical guide for applied research.

[27] Mayring P (2002). Qualitative social research.

[28] Unipark. Available from: https://www.unipark.com/ [Accessed 04 February 2019].

[29] Ravdin PM, Siminoff LA, Davis GJ, Mercer MB, Hewlett J, Gerson N (2001). Computer program to assist in making decisions about adjuvant therapy for women with early breast cancer. J Clin Oncol.

[30] Wishart GC, Bajdik CD, Dicks E, Provenzano E, Schmidt MK, Sherman M (2012). PREDICT plus: development and validation of a prognostic model for early breast cancer that includes HER2. Br J Cancer.

[31] Wishart GC, Azzato EM, Greenberg DC, Rashbass J, Kearins O, Lawrence G (2010). PREDICT: a new UK prognostic model that predicts survival following surgery for invasive breast cancer. Breast Cancer Res.

[32] Michaelson JS, Chen LL, Bush D, Fong A, Smith B, Younger J (2011). Improved web-based calculators for predicting breast carcinoma outcomes. Breast Cancer Res Treat.

[33] Haybittle JL, Blamey RW, Elston CW, Johnson J, Doyle PJ, Campbell FC (1982). A prognostic index in primary breast cancer. Br J Cancer.

[34] Campbell HE, Gray AM, Harris AL, Briggs AH, Taylor MA (2010). Estimation and external validation of a new prognostic model for predicting recurrence-free survival for early breast cancer patients in the UK. Br J Cancer.

[35] Kindts I, Laenen A, Peeters S, Janssen H, Depuydt T, Nevelsteen I (2016). Validation of the web-based IBTR! 2.0 nomogram to predict for ipsilateral breast tumor recurrence after breast-conserving therapy. Int J Radiat Oncol Biol Phys.

[36] Jones B (2012). BresDex: helping women make breast cancer surgery choices. J Vis Commun Med.

[37] Lundin J, Lundin M, Isola J, Joensuu H (2004). Validation of a web-based prognostic system for breast cancer. Stud Health Technol Inform.

[38] Van Zee KJ, Manasseh D-ME, Bevilacqua JLB, Boolbol SK, Fey JV, Tan LK (2003). A nomogram for predicting the likelihood of additional nodal metastases in breast cancer patients with a positive sentinel node biopsy. Ann Surg Oncol.

[39] Hassett MJ, O’Malley AJ, Pakes JR, Newhouse JP, Earle CC (2006). Frequency and cost of chemotherapy-related serious adverse effects in a population sample of women with breast cancer. J Natl Cancer Inst.

[40] Barcenas CH, Niu J, Zhang N, Zhang Y, Buchholz TA, Elting LS (2014). Risk of hospitalization according to chemotherapy regimen in early-stage breast cancer. J Clin Oncol.

[41] Campbell H, Taylor M, Harris A, Gray A (2009). An investigation into the performance of the adjuvant! Online prognostic programme in early breast cancer for a cohort of patients in the United Kingdom. Br J Cancer.

[42] de Glas NA, van de Water W, Engelhardt EG, Bastiaannet E, de Craen AJM, Kroep JR (2014). Validity of adjuvant! Online program in older patients with breast cancer: a population-based study. Lancet Oncol.

[43] Hajage D, de Ryke Y, Bollet M, Savignoni A, Caly M, Pierga J-Y (2011). External validation of Adjuvant! Online breast cancer prognosis tool. Prioritising recommendations for improvement. PLoS One.

[44] Jung M, Choi EH, Nam CM, Rha SY, Jeung HC, Lee SH (2013). Application of the adjuvant! Online model to Korean breast cancer patients: an assessment of prognostic accuracy and development of an alternative prognostic tool. Ann Surg Oncol.

[45] Yao-Lung K, Dar-Ren C, Tsai-Wang C (2012). Accuracy validation of adjuvant! Online in Taiwanese breast cancer patients--a 10-year analysis. BMC Med Inform Decis Mak..

[46] Mook S, Schmidt MK, Rutgers EJ, van de Velde AO, Visser O, Rutgers SM (2009). Calibration and discriminatory accuracy of prognosis calculation for breast cancer with the online adjuvant! Program: a hospital-based retrospective cohort study. Lancet Oncol.

[47] Olivotto IA, Bajdik CD, Ravdin PM, Speers CH, Coldman AJ, Norris BD (2005). Population-based validation of the prognostic model ADJUVANT! For early breast cancer. J Clin Oncol.

[48] Engelhardt EG, van den Broek AJ, Linn SC, Wishart GC, Rutgers EJT, van de Velde AO (2017). Accuracy of the online prognostication tools PREDICT and adjuvant! For early-stage breast cancer patients younger than 50 years. Eur J Cancer.

[49] Hearne BJ, Teare MD, Butt M, Donaldson L (2015). Comparison of Nottingham prognostic index and adjuvant online prognostic tools in young women with breast cancer: review of a single-institution experience. BMJ Open.

[50] Quintyne KI, Woulfe B, Coffey JC, Gupta RK (2013). Correlation between Nottingham prognostic index and adjuvant! Online prognostic tools in patients with early-stage breast cancer in mid-Western Ireland. Clin Breast Cancer.

[51] Plakhins G, Irmejs A, Gardovskis A, Subatniece S, Liepniece-Karele I, Purkalne G (2013). Underestimated survival predictions of the prognostic tools adjuvant! Online and PREDICT in BRCA1-associated breast cancer patients. Familial Cancer.

[52] Wishart GC, Bajdik CD, Azzato EM, Dicks E, Greenberg DC, Rashbass J (2011). A population-based validation of the prognostic model PREDICT for early breast cancer. Eur J Surg Oncol.

[53] de Glas NA, Bastiaannet E, Engels CC, de Craen AJM, Putter H, van de Velde CJH (2016). Validity of the online PREDICT tool in older patients with breast cancer: a population-based study. Br J Cancer.

[54] Maishman T, Copson E, Stanton L, Gerty S, Dicks E, Durcan L (2015). An evaluation of the prognostic model PREDICT using the POSH cohort of women aged ⩽40 years at breast cancer diagnosis. Br J Cancer.

[55] Wishart GC, Rakha E, Green A, Ellis I, Ali HR, Provenzano E (2014). Inclusion of KI67 significantly improves performance of the PREDICT prognostication and prediction model for early breast cancer. BMC Cancer.

[56] Wong H-S, Subramaniam S, Alias Z, Taib NA, Ho G-F, Ng C-H (2015). The predictive accuracy of PREDICT: a personalized decision-making tool for southeast Asian women with breast cancer. Medicine (Baltimore).

[57] Candido Dos Reis FJ, Wishart GC, Dicks EM, Greenberg D, Rashbass J, Schmidt MK (2017). An updated PREDICT breast cancer prognostication and treatment benefit prediction model with independent validation. Breast Cancer Res.

[58] Chen LL, Nolan ME, Silverstein MJ, Mihm MC, Sober AJ, Tanabe KK (2009). The impact of primary tumor size, lymph node status, and other prognostic factors on the risk of cancer death. Cancer.

[59] Laas E, Mallon P, Delomenie M, Gardeux V, Pierga J-Y, Cottu P (2015). Are we able to predict survival in ER-positive HER2-negative breast cancer? A comparison of web-based models. Br J Cancer.

[60] Alba AC, Agoritsas T, Walsh M, Hanna S, Iorio A, Devereaux PJ (2017). Discrimination and calibration of clinical prediction models: users’ guides to the medical literature. JAMA..

[61] Peele PB, Siminoff LA, Xu Y, Ravdin PM (2005). Decreased use of adjuvant breast cancer therapy in a randomized controlled trial of a decision aid with individualized risk information. Med Decis Mak.

[62] Siminoff LA, Gordon NH, Silverman P, Budd T, Ravdin PM (2006). A decision aid to assist in adjuvant therapy choices for breast cancer. Psychooncology.

[63] Aebi S, Sun Z, Braun D, Price KN, Castiglione-Gertsch M, Rabaglio M (2011). Differential efficacy of three cycles of CMF followed by tamoxifen in patients with ER-positive and ER-negative tumors: long-term follow up on IBCSG trial IX. Ann Oncol.

[64] Karlsson P, Sun Z, Braun D, Price KN, Castiglione-Gertsch M, Rabaglio M (2011). Long-term results of international breast Cancer study group trial VIII: adjuvant chemotherapy plus goserelin compared with either therapy alone for premenopausal patients with node-negative breast cancer. Ann Oncol.

[65] International Breast Cancer Study Group (1997). Effectiveness of adjuvant chemotherapy in combination with tamoxifen for node-positive postmenopausal breast cancer patients. J Clin Oncol.

[66] Fisher B, Dignam J, Wolmark N, DeCillis A, Emir B, Wickerham DL (1997). Tamoxifen and chemotherapy for lymph node-negative, estrogen receptor-positive breast cancer. J Natl Cancer Inst.

[67] Fisher B, Costantino J, Redmond C, Poisson R, Bowman D, Couture J (1989). A randomized clinical trial evaluating tamoxifen in the treatment of patients with node-negative breast cancer who have estrogen-receptor-positive tumors. N Engl J Med.

[68] Fisher B, Jeong J-H, Bryant J, Anderson S, Dignam J, Fisher ER (2004). Treatment of lymph-node-negative, oestrogen-receptor-positive breast cancer: long-term findings from National Surgical Adjuvant Breast and bowel project randomised clinical trials. Lancet..

[69] Hutchins LF, Green SJ, Ravdin PM, Lew D, Martino S, Abeloff M (2005). Randomized, controlled trial of cyclophosphamide, methotrexate, and fluorouracil versus cyclophosphamide, doxorubicin, and fluorouracil with and without tamoxifen for high-risk, node-negative breast cancer: treatment results of intergroup protocol INT-0102. J Clin Oncol.

[70] Early Breast Cancer Trialists’ Collaborative Group (EBCTCG). Comparisons between different polychemotherapy regimens for early breast cancer: meta-analyses of long-term outcome among 100 000 women in 123 randomised trials. Lancet 2012;379(9814):432–444. 10.1016/S0140-6736(11)61625-5 PMC3273723 22152853

[71] Early Breast Cancer Trialists’ Collaborative Group (EBCTCG). Effects of chemotherapy and hormonal therapy for early breast cancer on recurrence and 15-year survival: an overview of the randomised trials. Lancet 2005;365(9472):1687–1717. 10.1016/S0140-6736(05)66544-0 15894097

[72] Lord S, Ghersi D, Gattellari M, Wortley S, Wilcken N, Simes J (2004). Antitumour antibiotic containing regimens for metastatic breast cancer. Cochrane Database Syst Rev.

[73] Ferguson T, Wilcken N, Vagg R, Ghersi D, Nowak AK (2007). Taxanes for adjuvant treatment of early breast cancer. Cochrane Database Syst Rev.

[74] Ghersi D, Willson ML, Chan MMK, Simes J, Donoghue E, Wilcken N (2015). Taxane-containing regimens for metastatic breast cancer. Cochrane Database Syst Rev.

[75] Early Breast Cancer Trialists’ Collaborative Group (EBCTCG). Aromatase inhibitors versus tamoxifen in early breast cancer: patient-level meta-analysis of the randomised trials. Lancet 2015;386(10001):1341–1352. 10.1016/S0140-6736(15)61074-1 26211827

[76] Federal Statistical Office. [Life table Germany 2010/12]. Wiesbaden; 2015.

[77] Patnaik JL, Byers T, Diguiseppi C, Denberg TD, Dabelea D (2011). The influence of comorbidities on overall survival among older women diagnosed with breast cancer. J Natl Cancer Inst.

[78] Kiderlen M, de Glas NA, Bastiaannet E, van de Water W, de Craen AJM, Guicherit OR (2014). Impact of comorbidity on outcome of older breast cancer patients: a FOCUS cohort study. Breast Cancer Res Treat.

[79] Khullar D, Jena AB (2016). Reducing prognostic errors: a new imperative in quality healthcare. BMJ.

[80] Wilson PW, D’Agostino RB, Levy D, Belanger AM, Silbershatz H, Kannel WB (1998). Prediction of coronary heart disease using risk factor categories. Circulation.

[81] Shachar SS, Muss HB (2016). Internet tools to enhance breast cancer care. NPJ Breast Cancer.

[82] El Hage CH, Wazir U, Mokbel K, Kasem A, Mokbel K (2018). Do online prognostication tools represent a valid alternative to genomic profiling in the context of adjuvant treatment of early breast cancer? A systematic review of the literature. Am J Surg.

[83] Sparano JA, Gray RJ, Makower DF, Pritchard KI, Albain KS, Hayes DF (2018). Adjuvant chemotherapy guided by a 21-gene expression assay in breast Cancer. N Engl J Med.

[84] Orucevic A, Bell JL, McNabb AP, Heidel RE (2017). Oncotype DX breast cancer recurrence score can be predicted with a novel nomogram using clinicopathologic data. Breast Cancer Res Treat.

