# Supplementary material for: Communicating prognosis to women with early breast cancer – overview of prediction tools and the development and pilot testing of a decision aid
Source: BMC Health Serv Res. 2019 Mar 15;19:171. doi: 10.1186/s12913-019-3988-2 (PMC6420759; doi:10.1186/s12913-019-3988-2)
Supplement: Supplementary file 1 — Table S1. 1. Overview of excluded tools for breast cancer patients. Table 1 contains an overview of other tools for breast cancer patients that did not meet our inclusion criteria. 2. Search strategy adjuvant therapy in PubMed. The search strategy listed here was used to search for studies directly comparing endocrine therapy to chemoendocrine therapy in PubMed. 3. Main content of the decision aid. This is the English translation of the table of contents of the decision aid. 4. Interview guide: Focus group interviews SPUPEO DA drug therapy. This is the English translation of the interview guide used for focus group interviews. (DOCX 27 kb) [file 12913_2019_3988_MOESM1_ESM.docx]

**Additional file 1: Table S1 Overview of excluded tools for breast cancer patients**

| **Tool** | **Reason for exclusion** |
| --- | --- |
| Nottingham Prognostic Index | Nodal involvement, nodal size and grade only, no patient characteristics |
| Options | Effect of adjuvant drug therapy on recurrence, not survival (no longer available) |
| IBTR | Effect of radiotherapy on 10-year ipsilateral breast tumor recurrence, not survival |
| MD Anderson clinical calculators  <https://www.mdanderson.org/for-physicians/clinical-tools-resources/clinical-calculators.html> | Effect of neoadjuvant chemotherapy on residual cancer burden, disease-free survival and chemotherapy response as well as benefit of radiation for older patients with conservative surgery |
| BresDex | Surgery for early breast cancer, currently off-line |
| Finprog | Case-match survival estimation based on prognostic factors |
| Memorial Sloan Kettering Cancer Center breast cancer nomogram | DCIS recurrence and nodal metastasis |

**2. Search strategy adjuvant therapy in PubMed**

(((breast neoplasms[MeSH Terms]) AND ((adjuvant chemotherapy[MeSH Terms] OR (antineoplastic combined chemotherapy protocols[MeSH Terms]) OR (anthracyclines[MeSH Terms] OR taxoids[MeSH Terms] OR antimetabolites, antineoplastic[MeSH Terms] OR alkylating antineoplastic agents[MeSH Terms]))))) OR (((breast neoplasm* OR breast tumor* OR human mammary neoplasm* OR human mammary carcinoma* OR Breast Cancer and breast carcinoma) AND (((adjuvant chemotherapy OR adjuvant drug therapy) OR (antineoplastic combined chemotherapy protocol* OR cancer chemotherapy protocol* OR combined antineoplastic agents OR antineoplastic drug combinations OR anticancer drug combinations)) OR ((anthracycline* OR epirubicin OR farmorubicin OR doxorubicin* OR adriamycin) OR (taxoid* OR taxan* OR paclitaxel OR taxol OR docetax* OR taxotere) OR (antineoplastic antimetabolite* OR 5-FU OR 5-Fluorouracil OR Fluorouracil OR methotrexate) OR (alkylating antineoplastic agent* OR cyclophospha*)))))

**3. Main content of the decision aid**

Important information for readers about the decision aid:

Where does the information in this brochure come from?

Shared decision-making – how does it work?

What is breast cancer?

What is hormone receptor-positive breast cancer?

My report

What are the treatment goals?

Adjuvant therapy: What are the drug treatment options?

What is the benefit of drug treatment?

What is the harm of adjuvant chemotherapy?

What is the harm of treatment with tamoxifen?

Where do you find further information and support?

**Appendix – Knowing more**

What is the female breast made of?

Which information will you find in your report?

What is the chance that a woman will die from breast cancer?

Is biomarker-testing helpful for treatment decisions?

Antihormonal therapy

Portacath (port)

Can you influence the course of the disease yourself?

Dictionary

Literature used in this decision aid

Who developed this decision aid?

**4. Interview guide: Focus group interviews SPUPEO DA drug therapy**

**Introduction (10 min.)**

- Welcome: Introduction of the interviewer and the department of health sciences and education, Hamburg University. Explanation of background and aim of the SPUPEO study
- Written informed consent to participate in the study, data privacy statement and consent to audio recording
- Aim of the focus group interview, explanation on how the interview will be carried out
- Reminder: Please switch off your mobile phone
- Information: ”Please help yourself to beverages and biscuits”
- Information: “The allowance of 15€ will be given to participants after the focus group interview”

**Warm - up (30 min.)**

- Introduction of the participants
- Coming here, what things went through your mind?
- First thoughts and impressions on the decision aid („think aloud“ method). What went through your mind when you flicked through the DA for the first time?

**Main questions: Evaluation of the DA (60 min.)**

As you know/read/were being told, this focus group interview is on the new DA for women with breast cancer. You received the DA by mail and we would like to discuss it with you.

**A Clarity**

1.) Importance of comorbidities

(p. 28/29) Participants comprehension of the importance of the comorbidities.

Please imagine you are telling a friend about it. How would you explain it to her in your own words?

2.) Presentation

Participants comprehension of the bar charts (p. 12/13) and the presentation on adverse events (p. 18/19)

Think aloud method

3.) Uncertainty concerning the evidence

- Participants comprehension of…

… the insufficient evidence concerning the direct comparison of endocrine therapy to the combination of chemotherapy and endocrine therapy?

Please imagine you are telling a friend about it. How would you explain it to her in your own words?

4.) Evidence base / background of the decision aid

• Participants comprehension of…

… the scientific approach of the DA?

… what is evidence-based information?

… aim and objective of the decision aid?

Please imagine you are telling a friend about it. How would you explain it to her in your own words?

5.) General aspects – readability and clarity

- Which part of the decision aid is more difficult to read/understand?
- Is the structure comprehensible and well done (concerning the outline as well as the structure of the individual chapters. Is the dichotomy of the DA comprehensible?)?

6.) Input from the participants

- Is there anything missing, any content you would like to add? What did you notice? What was difficult for you? What would you like to add to the DA?

**B Relevance**

1.) Topics

- Have all important topics been addressed in the DA? Do you miss anything?

**Farewell**

Thank you for participating

Allowance
